# Use of the CPD-REACTION Questionnaire to Evaluate Continuing Professional Development Activities for Health Professionals: Systematic Review

**DOI:** 10.2196/36948

**Published:** 2022-05-02

**Authors:** Gloria Ayivi-Vinz, Felly Bakwa Kanyinga, Lysa Bergeron, Simon Décary, Évèhouénou Lionel Adisso, Hervé Tchala Vignon Zomahoun, Sam J Daniel, Martin Tremblay, Karine V Plourde, Sabrina Guay-Bélanger, France Légaré

**Affiliations:** 1 VITAM – Centre de Recherche en Santé Durable, Centre Intégré Universitaire de Santé et de Services Sociaux de la Capitale-Nationale Université Laval Quebec, QC Canada; 2 Tier 1 Canada Research Chair in Shared Decision Making and Knowledge Translation Université Laval Quebec, QC Canada; 3 Department of Social and Preventive Medicine, Faculty of Medicine Université Laval Quebec, QC Canada; 4 School of Rehabilitation, Faculty of Medicine and Health Sciences Université de Sherbrooke Sherbrooke, QC Canada; 5 Unité de Soutien SSA Québec Université Laval Quebec, QC Canada; 6 Direction du Développement Professionnel Continu, Fédération des Médecins Spécialistes du Québec Montreal, QC Canada; 7 Department of Family Medicine and Emergency Medicine, Faculty of Medicine Université Laval Quebec, QC Canada

**Keywords:** CPD-REACTION, behavior, intention, education medical, continuing, health care professionals, questionnaire, web-based, continuing professional development

## Abstract

**Background:**

Continuing professional development (CPD) is essential for physicians to maintain and enhance their knowledge, competence, skills, and performance. Web-based CPD plays an essential role. However, validated theory–informed measures of their impact are lacking. The CPD-REACTION questionnaire is a validated theory–informed tool that evaluates the impact of CPD activities on clinicians’ behavioral intentions.

**Objective:**

We aimed to review the use of the CPD-REACTION questionnaire, which measures the impact of CPD activities on health professionals’ intentions to change clinical behavior. We examined CPD activity characteristics, ranges of intention, mean scores, score distributions, and psychometric properties.

**Methods:**

We conducted a systematic review informed by the Cochrane review methodology. We searched 8 databases from January 1, 2014, to April 20, 2021. Gray literature was identified using Google Scholar and Research Gate. Eligibility criteria included all health care professionals, any study design, and participants’ completion of the CPD-REACTION questionnaire either before, after, or before and after a CPD activity. Study selection, data extraction, and study quality evaluation were independently performed by 2 reviewers. We extracted data on characteristics of studies, the CPD activity (eg, targeted clinical behavior and format), and CPD-REACTION use. We used the Mixed Methods Appraisal Tool to evaluate the methodological quality of the studies. Data extracted were analyzed using descriptive statistics and the Student t test (2-tailed) for bivariate analysis. The results are presented as a narrative synthesis reported according to the PRISMA (Preferred Reporting Items for Systematic Reviews and Meta-Analyses) guidelines.

**Results:**

Overall, 65 citations were eligible and referred to 52 primary studies. The number of primary studies reporting the use of CPD-REACTION has increased continuously since 2014 from 1 to 16 publications per year (2021). It is available in English, French, Spanish, and Dutch. Most of the studies were conducted in Canada (30/52, 58%). Furthermore, 40 different clinical behaviors were identified. The most common CPD format was e-learning (34/52, 65%). The original version of the CPD-REACTION questionnaire was used in 31 of 52 studies, and an adapted version in 18 of 52 studies. In addition, 31% (16/52) of the studies measured both the pre- and postintervention scores. In 22 studies, CPD providers were university-based. Most studies targeted interprofessional groups of health professionals (31/52, 60%).

**Conclusions:**

The use of CPD-REACTION has increased rapidly and across a wide range of clinical behaviors and formats, including a web-based format. Further research should investigate the most effective way to adapt the CPD-REACTION questionnaire to a variety of clinical behaviors and contexts.

**Trial Registration:**

PROSPERO CRD42018116492; https://www.crd.york.ac.uk/prospero/display_record.php?RecordID=116492

## Introduction

Continuing professional development (CPD) encompasses the multiple educational and developmental activities that health care professionals undertake to maintain and enhance their knowledge, skills, performance, and relationships in the provision of health care. The ultimate goal of CPD is to enhance the quality and safety of patient care and enhance both patient experience and health outcomes [1]. In recent years, web-based CPD has increased exponentially, and the recent COVID-19 pandemic has emphasized the need for more effective web-based CPD. Health professional behavior change (adoption or abandonment of a practice) is a long and complex process [2]. The Kirkpatrick model conceptualizes a framework for CPD assessment that measures four distinct outcome levels: satisfaction; knowledge, skills, or attitudes; transfer of learning to practice (ie, behavior); and organizational outcomes such as productivity and quality [3].

The lack of validated instruments informed by behavior change theories for assessing CPD outcomes has slowed the advancement of the CPD knowledge base [4]. In 2011, a consortium of CPD providers from the Province of Quebec, Canada, developed a tool to assess Kirkpatrick level 3 outcomes (transfer of learning to practice) based on an integrated model explaining behavior change among health professionals [5,6]. This model posits that intention is a strong predictor of behavior, and that behavioral intention, in turn, is influenced by beliefs about capabilities, beliefs about consequences, moral norms, and social influences [5]. The resulting tool, the CPD-REACTION questionnaire, is a comprehensive, theory-based, validated instrument for assessing the impact of accredited CPD activities on clinical behavioral intention [7,8]. During the past 10 years, it has been used in regular evaluations of the effects of CPD activities on behavior change by major CPD providers such as the Federation of Medical Specialists of Quebec (Fédération des Médecins Spécialistes du Québec) and to assess training for a wide variety of other health care professionals [9-12].

However, the current range of CPD-REACTION use remains unknown. Moreover, the clinical topics of CPD activities evaluated using the tool, the types of clinical behaviors sought, how often it has been used to evaluate web-based CPD, what kind of health care professionals are targeted by such CPD activities, and how the results shown by CPD-REACTION in terms of behavior change intentions are used, are also unknown. Although tool validity has been demonstrated in the Canadian context [8], other evidence on its cross-cultural validity and psychometric properties is still lacking. Therefore, we aimed to systematically review studies that have used the CPD-REACTION questionnaire.

Our research questions were as follows: (1) What are the characteristics of CPD activities in studies using CDP-REACTION? (2) What are the ranges of behavioral change intentions, mean scores, and distribution of scores across all studies that used CPD-REACTION? (3) What are the psychometric properties of CPD-REACTION?

## Methods

### Ethics Approval and Consent to Participate

As this research was based on published studies, ethics approval was not required for this systematic review. The protocol was registered in the PROSPERO (International Prospective Register of Systematic Reviews) registry under the number CRD42018116492 on December 4, 2018. The main change to the protocol was the inclusion of references to studies reported in a language other than English and French.

### Study Design

Informed by the Cochrane review methodology [13], we conducted a systematic review and followed the PRISMA (Preferred Reporting Items for Systematic Reviews and Meta-Analyses) 2020 statement [14].

### Eligibility Criteria

Informed by the PICOS (Population, Intervention, Comparator, Outcomes, Study design) model [15], the inclusion criteria were as follows: (1) *Population*- the target population considered for this review included all individuals working in health fields who completed an original, translated, or adapted version of the CPD-REACTION questionnaire before, after, or before and after an activity. There was no age restriction or restriction of health care professions (eg, physician, nurse, or any other health professional). They could be working in the public or private sector, in the process of training, or have already graduated; (2) *Intervention-* not specified; (3) *Comparator*- not specified; (4) *Outcomes*- The original, adapted, or translated version of the 12-item CPD-REACTION was used to assess the intention to change either a clinical practice or a health behavior. On the basis of the Godin integrated model, this tool is a questionnaire composed of 12 items that measure behavioral intention and some of its predictors; that is, beliefs about capabilities, beliefs about consequences, moral norms, and social influences [5,7]. The 5 constructs of the CPD-REACTION questionnaire have been validated, with Cronbach coefficients for the constructs varying from 0.77 to 0.85 [7,8]; (5) *Study Design*-Any study design was considered: randomized clinical trials (individual, group, or cluster, including stepped-wedge), before-and-after studies, translation studies, ecology studies, qualitative studies, or any mixed-study design (if they included the use of CPD-REACTION). Only primary studies were considered for inclusion in this systematic review. Therefore, we did not include any systematic reviews. These articles could be reported in any language.

### Information Sources

The literature search was performed using eight databases: Embase, MEDLINE/PubMed, Web of Science, ERIC-EBSCO, PsycINFO-ovid, CINAHL, Social Sciences Full Text-EBSCO, and Academic Search Premier EBSCO. A temporal filter was applied from January 1, 2014, to April 20, 2021, because CPD-REACTION was published in 2014 [7]. We also performed a forward citation search using Google Scholar and Research Gate to identify studies citing the 3 main studies on the development and validation of CPD-REACTION (Multimedia Appendix 1).

### Search Strategy

The first phase of developing the search strategy was carried out on PubMed and reviewed by the authors to ensure that the concepts covered all research questions. This strategy was then translated into expressions that were adapted to each database. A documentary research expert revised the search strategy and the final version was based on three key concepts: “continuing education,” “CPD-REACTION questionnaire,” and “questionnaires.” These key concepts were searched using a combination of controlled vocabulary (MeSH [Medical Subject Headings] terms) and free-text search queries (Multimedia Appendix 1).

### Selection Process

Duplicates were identified using EndNote ×9 [16] and manual checking. First, reviewers (GA-V, FBK, LB, and LS) performed an independent selection based on the title and abstract. Second, all relevant references were considered for selection by full text (GA-V, FBK, LL, LB, and LS). An internet-based system, Covidence [17], was used to complete this step. The 2 reviewers then discussed and resolved any disagreement to obtain a consensus on study selection according to the eligibility criteria and, if necessary, consulted a third author (KVP). The reasons for exclusion of articles were documented.

### Data Extraction

A coding guide and corresponding extraction grid were developed and tested by the reviewers. The reviewers (GA-V, FBK, LL, LB, and LS) individually extracted data from the included studies. The reviewers discussed and resolved any disagreement.

Qualitative and quantitative data were extracted. The main groups of variables were (1) study characteristics, including author names, study design, study objectives, country, and type of CPD activity; (2) characteristics of the study participants, such as profession, setting (eg, hospital or university), average age, sex, study population (eg, single profession, mixed professions, and patients included); (3) CPD activity characteristics, such as country in which it was used, health field, duration of CPD activity, when tool was used (eg, pre- or post-CPD activity), format of CPD activity (eg, web-based), title of CPD activity, clinical behavior change targeted; (4) CPD-REACTION version used (original or adapted), adaptations to the questionnaire, eg, translations; (5) score values (mean, median, SD, minimum, and maximum) for all constructs measured, that is, behavioral intention, beliefs about capabilities, social influence, moral norm, and beliefs about consequences; (6) psychometric properties (Cronbach *α*, *κ*, or Cohen *d*).

### Methodological Quality Assessment of Individual Studies

Two examiners (GA-V and FBK) assessed the quality of each identified study using the Mixed Methods Appraisal Tool (MMAT), a validated tool for evaluating the quality of qualitative, quantitative, and mixed methods studies [18]. For each type of study design, 5 criteria were evaluated and each was rated “yes,” “can’t tell,” or “no.” The tool guideline discourages the calculation of an overall score, instead suggesting presenting detailed ratings for each criterion [18] (Multimedia Appendix 2).

### Data Synthesis

Given the large variety of behavior changes targeted by studies (clinical practice behaviors and others) and the methodological and statistical heterogeneity of studies, we performed a narrative synthesis using descriptive statistics. For the CPD-REACTION score values, we did not calculate the average scores for the construct if CPD-REACTION did not evaluate the same behavior. Instead, we summarized the construct scores based on the timing of the evaluation, that is, if it was a pre-post, only pre-evaluation, or only postevaluation. Descriptive statistics were computed using STATA (version 11; StataCorp). To summarize the target behaviors of the included studies, we performed a thematic analysis. After the analysis, we organized and summarized the main behaviors based on the emerging themes, namely, “shared decision-making,” “decision aids or toolkit,” and “others.”

## Results

### Study Selection

**Figure 1:**
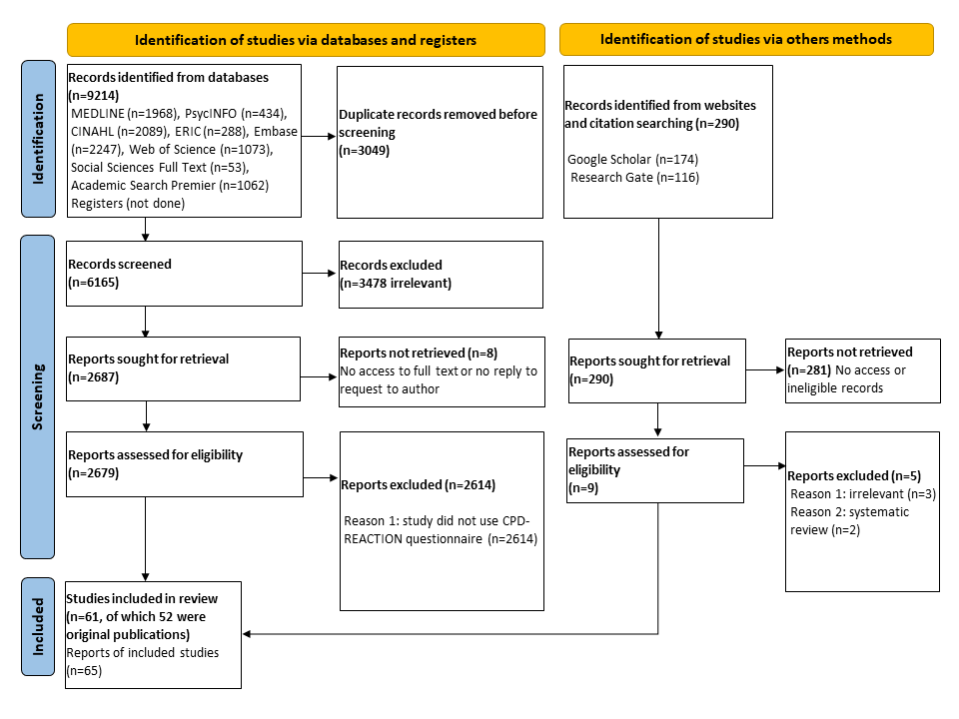
PRISMA (Preferred Reporting Items for Systematic Reviews and Meta-Analyses) 2020 flowchart.

We described the selection process in a PRISMA flowchart (Figure 1). A total of 9504 records were identified and 3330 (duplicates or ineligible) were removed. After screening, 65 records matched the eligibility criteria and referred to 61 publications and 52 unique studies [7-12,19-70] (Figure 1).

### Study Characteristics

**Figure 2:**
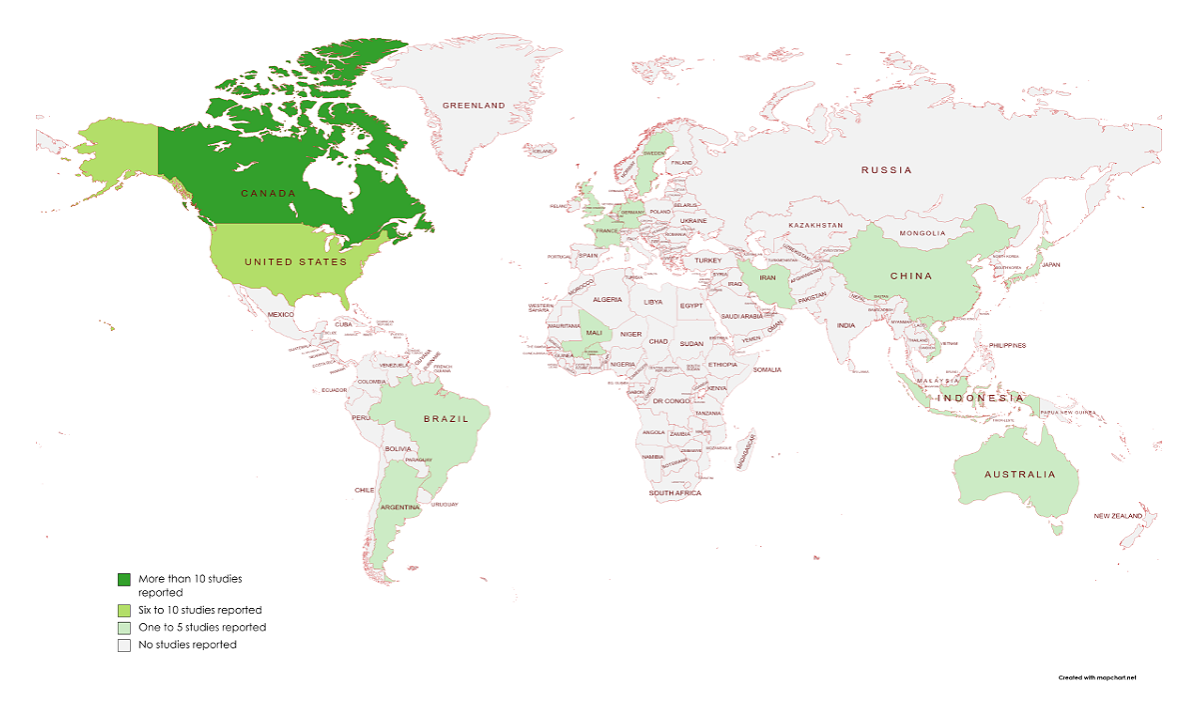
Distribution of published studies worldwide that used the CPD-REACTION questionnaire.

Since 2014, the number of published studies using CPD-REACTION has increased from 1 to 16 publications in 2021 (Multimedia Appendix 3). Of all the studies, 69% (36/52) were published between 2019 and 2021 [9,32-68]. Furthermore, 58% (30/52) were located in Canada [7,8,10-12,23-25,28,31,35,38-41,44-51,53,54,56,60,63,65,67] and the rest in the United States (n=6), the United Kingdom (n=4), Australia (n=2) [30,34], Iran (n=2) [55,64], Argentina (n=1) [52], Indonesia (n=1) [32], Germany (n=1) [58], Sweden (n=1) [21], the Netherlands (n=1) [59], and Burkina Faso (n=1) [37]. In addition, two multicountry studies were reported: 1 from Brazil-China-France-Japan-Mali [68] and 1 from Canada-Vietnam [19] (Table 1; Figure 2). There were no exclusive qualitative studies (Table 1). Most study designs were mixed methods (24/52, 46%) [8,9,12,18,23,26,35,42,​^43^,^45^,^47^,^50^,^55^,^56^,^61^,^64^,^67^,^71^,^72^], followed by cross-sectional studies (9/52, 17%) [22,32,39,42,56,59].

**Table 1:** Study and intervention characteristics (N=52).

Study and intervention characteristics		Number of studies, n (%^a^)
**Study location**		
	Canada	30 (58)
	United States	6 (12)
	United Kingdom	4 (8)
	Australia	2 (4)
	Iran	2 (4)
	Argentina	1 (2)
	Burkina Faso	1 (2)
	Germany	1 (2)
	Indonesia	1 (2)
	Netherlands	1 (2)
	Sweden	1 (2)
	Canada and Vietnam	1 (2)
	China-Brazil-France-Mali-Canada-Japan	1 (2)
**Study design**		
	Mixed methods study	24 (46)
	Cross-sectional study	9 (17)
	Baseline and follow-up or before-after or comparative study	7 (13)
	Randomized trial	4 (8)
	Quasi-experimental study	3 (6)
	Validation study	2 (4)
	Cohort study	2 (4)
	Intervention study	1 (2)
**Clinical setting**		
	Multicenter academic hospitals	3 (6)
	Multicenter community hospitals	11 (21)
	Multicenter both academic and community	11 (21)
	Single-center academic hospital	4 (8)
	Single-center community hospital	5 (10)
	Not a clinical setting	13 (25)
	Not reported or not applicable	5 (10)
**Type of CPD^b^ activities**		
	Course or workshop	31 (60)
	Conference	1 (2)
	Other^c^ CPD activities	4 (8)
	No activity pertaining to CPD^d^	3 (6)
	Not specified or not applicable	13 (25)
**CPD activity Format**		
	Web-based	34 (65)
	In person	13 (25)
	Not specified	5 (10)
**Version of questionnaire used**		
	Adapted	18 (35)
	Original	31 (60)
	Not specified	3 (6)
**When CPD-REACTION was used**		
	Preactivity	6 (12)
	Postactivity	11 (21)
	Pre- and postactivity	16 (31)
	Not specified or not applicable	19 (37)
**Delivery mode of CPD-REACTION questionnaire**		
	Digital platform or web-based	19 (37)
	Web-based and paper	1 (2)
	Paper copy	16 (31)
	Not specified	16 (31)
**Language of CPD questionnaire used**		
	Dutch	1 (2)
	Spanish	1 (2)
	English	28 (54)
	French	14 (27)
	English and French	3 (6)
	Not reported	5 (10)
**Type of CPD provider**		
	Government	1 (2)
	Hospital	13 (25)
	Private company	6 (12)
	University	22 (42)
	Not specified or not applicable	7 (19)

^a^All percentages may not add up to 100%.

^b^CPD: continuing professional development.

^c^Training or workshop combined with activities such as face-to-face meetings, media interviews, minutes documenting interactions, conferenced meetings, annual national collaboration meeting, and team meeting to watch video.

^d^Guidelines application, outreach sessions.

### Characteristics of the Study Participants

**Figure 3:**
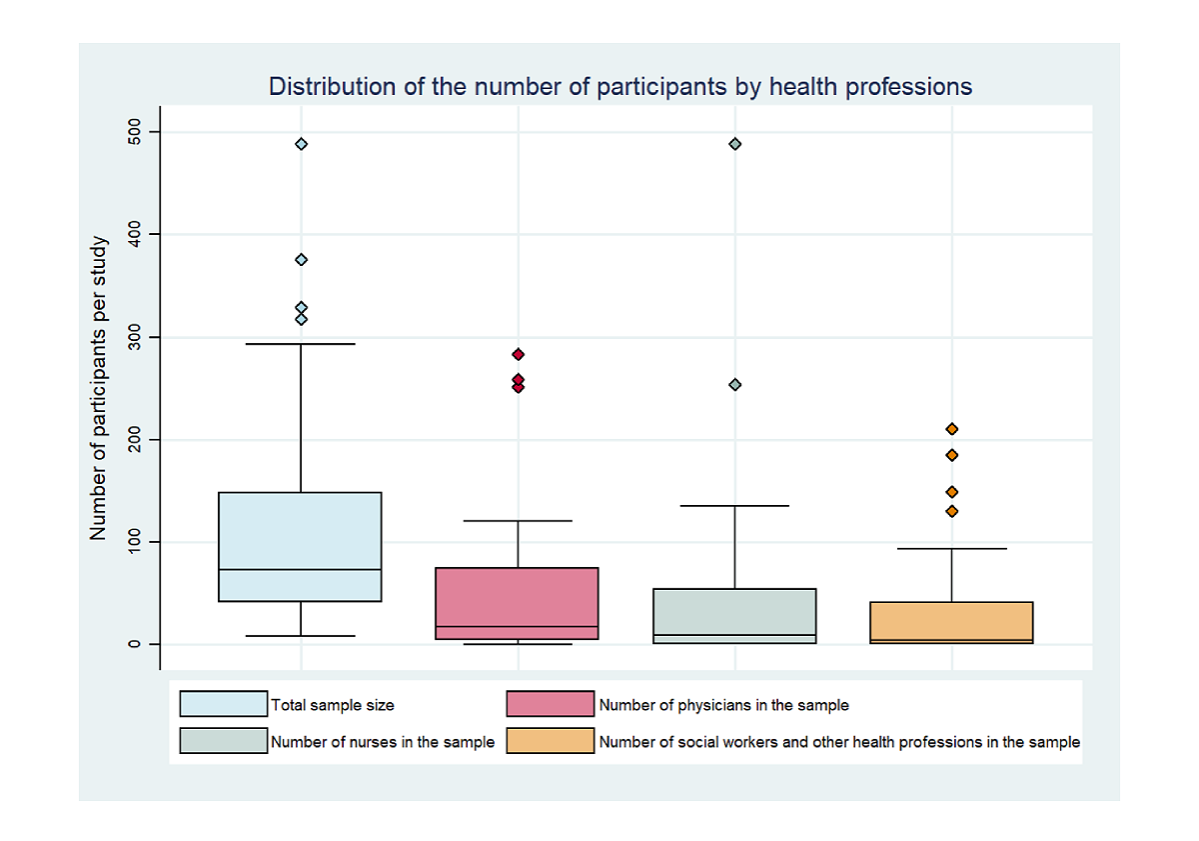
Boxplot of number of participants by health profession present at each continuing professional development activity.

In total, CPD-REACTION was administered to 4886 participants. Even when age was mentioned, it was not possible to properly report on age because of the heterogeneity of age ranges. The sex of the participants was not reported for all studies. The authors mostly defined participants based solely on their profession. Physicians were the most represented health profession (1843/4886, 37.72%). Furthermore, 7 studies included residents or unlicensed health professionals [8,36,38,39,46,55,64]. (Table 2). In most studies, participants in CPD activities consisted of interprofessional groups (30/52, 60%) [7,9-11,19,21,27,28,31,35,37,41,43,44,46,47,50,51,53, 55-61,63,64,66-68]. Professions included nurses (5/52, 10%) [26,29,30,48,54], physicians (4/52, 8%) [38,39,49,52], social workers or other health professionals, namely occupational therapists, physiotherapists, dietitians, behavioral counselors, nutritionists, health researchers (4/52, 8%) [23-25,34,36], specialist physicians (3/52, 6%) [32,40,65], and pharmacists (2/52, 4%) [22,42]. The presence of managers or decision-makers among participants was reported in 7 (13%) out of 52 studies. The number of participants per study ranged from 8 to 489 (Figure 3).

The largest proportion of CPD providers reported was university-based (22/52, 42%), whereas others were based in hospitals (13/52, 25%), private companies (6/52, 12%), or government (1/52, 2%).

**Table 2:** Professional profiles of study participants.

Population characteristics		Frequency, n (%^a^)
**Population of interest, in the studies (n=52)**		
	Physicians	8 (15.4)
	Interprofessional groups	31 (59.6)
	Nurses	5 (9.6)
	Other health professions^b^	8 (15.4)
**Number of participants per professional group (n=4886)**		
	Interprofessional groups	1843 (37.7)
	Nurses	1568 (32.1)
	Social workers and other health professionals	1053 (21.6)
	Not specified	422 (8.6)
**Presence of managers or decision-makers among participants (n=52)**		
	Yes	7 (13.5)
	No	45 (86.5)
**Presence of residents or unlicensed health professionals among participants (n=52)**		
	Yes	7 (13.5)
	No	45 (86.5)

^a^All percentages may not add up to 100%.

^b^Pharmacists, physical therapists, physiotherapists, providers of radiation therapy, midwives, and social workers.

### CPD Activity Characteristics

The questionnaire was administered in four languages: French (14/52, 27%) or English (28/52, 54%) [8-11,19,21-24,​^26^-^32^,^34^-^37^,^39^-^51^,^53^-^55^,^57^,^59^-^68^], Spanish (1/52, 2%) [52] and Dutch (1/52, 2%) [58]. The median number of CPD activities targeting behavior change per study was 1 and varied between 1 and 9 CPD activities per study. One-quarter (11/52, 21%) of the studies used CPD-REACTION to measure behavioral change intention but were not linked to a specific CPD activity [21,22,29,31,38,40,45,46,51,53,59] (Table 1). The most common format for CPD activities was web-based or e-learning based (34/52, 65%). The duration of CPD activities ranged from 30 to 225 minutes, with an average of 115 (SD 67) minutes.

### Targeted Clinical Behavior and Scoring of CPD-REACTION

The evaluations targeted 39 different clinical behaviors [7-12,19-70]. Thematic analysis showed that 7 (18%) out of the 39 pertained to shared decision-making [9,35,50,54,56,64,67] and 5 pertained to decision aids or toolkit implementation [53,60,61,63,66] (Table 3).

Regarding studies reporting mean scores after the intervention (n=33) [8,12,22,25,29,30,33-36,40-44,47-49,55,56,59,60,63,​^64^,^66^], 9 studies (27%) reported both pre- and postactivity scores [8,22,25,29,42,49,56,60,66]. The scores were all higher after the intervention. Furthermore, in all 9 studies, the pre-post score ranges (2.5-5.7) were higher than in studies measuring prescores only (2.6-5.2) or postscores only (1.8-4.8; Table 4). The average difference between the pre- and postintention scores was 0.54 SD 0.13. Among the 5 CPD-REACTION constructs, social influence scored the lowest (43; Table 4).

**Table 3:** Main behaviors targeted in included studies (n=39).

	Main clinical behavior targeted in included studies	Topic theme		
		SDM^a^	Decision aids or toolkit	Others
1.	To prescribe spirometry and to interpret the result [38,39]	−^b^	−	+^c^
2.	To actively engage with and invite patients who are underserved for Medicine Use Reviews (MURS) [20,42,43]	−	−	+
3.	To adopt SDM [53]	+	−	−
4.	To engage older patients living with dementia and their caregivers in decision-making about choosing a health intervention, based on the TPB [10]	+	−	−
5.	To use Decision Box to explain to patients the benefits and harms of the options, based on the TPB [10]	−	+	−
6.	To use a decision aid in clinical practice after completing the web-based program “MyDiabetesPlan” [63]	−	+	−
7.	To implement developmental coordination disorder (DCD) best practices [23]	−	−	+
8.	To provide medical abortion [40]	−	−	+
9.	To use COSTARS (pan-Canadian Oncology Symptom Triage and Remote Support) practice guides [45]	−	−	+
10.	To use of 15 evidence-informed symptom practice guides for providing telephone or in-home nursing services to clients with cancer [45]	−	−	+
11.	To engage in IP-SDM (interprofessional shared decision-making) [50]	+	−	−
12.	To use patient decision aids [12,56,64]	−	+	−
13.	To counsel patients regarding HIV prep therapy [22]	−	−	+
14.	To use IP-SDM [50,55]	+	−	−
15.	To apply the disclosure guidelines to my practice [44]	−	−	+
16.	To apply the Situation-Background-Assessment-Recommendation (SBAR) to my practice [44]	−	−	+
17.	To apply quality improvement strategies to solve challenges in my practice [44]	−	−	+
18.	To practice the person-centered approach (PCA—MACHIP 2) in maternal health [37]	−	−	+
19.	“Utiliser l’outil d’évaluation du risque de violence” (To use the Risk of Violence evaluation tool) [31]	−	−	+
20.	To collaboratively work with and actively involve children and young people who self-harm in their care [29]	−	−	+
21.	To use the evidence of implementing FREEDOM [46]	−	−	+
22.	To implement the STEADI toolkit [61]	−	+	−
23.	To report research translation and impact on the CV^d^ [51]	−	−	+
24.	To use SDM [35]	+	−	−
25.	To prescribe no pharmacological treatments [36]	−	−	+
26.	To use SDM with their next patient facing a preference-sensitive decision [56]	+	−	−
27.	To apply a systematic framework to identify and manage patients with dementia [34]	−	−	+
28.	To change and improve practice based on the interventions, that is, to order pneumococcal vaccines [41]	−	−	+
29.	To use research evidence in rheumatology [21]	−	−	+
30.	To successfully plan and implement evidence-based practice changes in health facility [27]	−	−	+
31.	To consider probiotic recommendation in infants and toddler patients [32]	−	−	+
32.	To perform SDM (action) among health professionals in any clinical setting [64]	+	−	−
33.	To use an app to decide about prenatal screening [9,54]	−	+	−
34.	To formulate a violence risk assessment and management plan [30]	−	−	+
35.	To use de-escalation techniques during escalating aggression [30]	−	−	+
36.	To use breakaway techniques when responding to a violent person [30]	−	−	+
37.	To change their practice about compassion fatigue education [57]	−	−	+
38.	To implement the 5A method training in the area of physical activity promotion [58]	−	−	+
39.	To care for children and young people admitted to hospital with self-harm [29]	−	−	+

^a^SDM: shared decision-making.

^b^Not related to theme.

^c^Related to theme.

^d^CV: Curriculum Vitae.

**Table 4:** Summary of pre- and postscores for all constructs of CPD-REACTION.

			Value, n		Pre-CPD^a^ activity (range)			Post-CPD activity (range)	
**Interventions with pre- and post-CPD scores**			9						
	Intention	9		4.5-6.5			5.7-6.8		
	Social influence	9		2.5-5.6			3.8-5.8		
	Beliefs about capabilities	9		3.2-6			5.4-6.4		
	Moral norm	7		5.51-6.7			6.2-6.9		
	Beliefs about consequences	9		5.73-6.6			6.2-6.8		
**Interventions with only prescores**									—^b^
	Intention	5		2.9-6.6					
	Social influence	5		2.6-6					
	Beliefs about capabilities	5		2.4-6.6					
	Moral norm	5		4.3-6.8					
	Beliefs about consequences	4		5.2-6.7					
**Interventions with only postscores**						—			
	Intention	19					3.4-7		
	Social influence	18					1.8-6.3		
	Beliefs about capabilities	18					3.9-6.8		
	Moral norm	18					4.6-6.9		
	Beliefs about consequences	18					4.8-4.8		

^a^CPD: continuing professional development.

^b^Author did not report or measure mean scores.

### CPD-REACTION Adaptations and Psychometric Properties

One-third (18/52, 35%) of the included studies reported having adapted CPD-REACTION [19,21,22,27,28,32,39,40,53,​^55^,^56^,^58^,^63^,^66^-^68^,^73^] (Table 1). Adaptations to the questionnaire reported were reformulated items (n=3) [19,28,49], using only certain construct scales (n=3 studies) [22,53,55], adding or dropping some items without reformulating the original items (n=3) [39,56,67], reporting percentages instead of score values ranging from 1 to 7 (n=2) [23,27], translation of the questionnaire into other languages (n=2) [52,58], and using a 5-point instead of 7-point Likert scale (n=1) [40]. Furthermore, more than 80% of all studies (48/52, 92%) reported the psychometric parameters of the original version of CPD-REACTION or else stated it was a validated tool [8,10,23,25,26,29-32,34-37,40-57,60,61,64-68,74]. In addition, 4 studies reported the psychometric properties of their adapted versions [28,39,52,69], with the Cronbach *α* of the included constructs ranging from 0.62 to 0.91.

### Risk of Bias in Studies

Although none of the studies fully met all MMAT criteria, none were rated “no” for any criteria (Multimedia Appendix 2). In the 4 quantitative randomized trials, only the criterion “randomization appropriately performed” was met by all 4 studies [10,50,54,63], and in all 17 mixed method studies, only the criterion “adequate rationale for using a mixed methods design” was met. In all the design groups, all the criteria not rated “yes” were rated “not sure.”

## Discussion

### Principal Findings

We found 61 publications of 52 unique studies that reported the use of the CPD-REACTION questionnaire to assess changes in behavioral intention among health professionals. Although the tool is aged <10 years (2014), we observed the most rapid increase in its use in the past 3 years, mostly in Canada, where it was developed. However, its use has spread to many other countries, including lower- and middle-income countries, and it is found in numerous languages (our finding of only 4 is an underrepresentation, as the team that produced the tool has agreed to translations into 8 languages) [75]. Since its inception, CPD-REACTION has been used by close to 5000 participants to target 39 clinical behaviors. The participants included 8 types of health professionals, with physicians and nurses being the most reported. Two-thirds of the studies included interprofessional clinical teams, including one in which 10 managers or decision-makers were CPD activity participants. The tool appeared to be mostly used for evaluating e-learning (n=34). In many cases, users adapted the questionnaire, such as using only certain construct scales or adding or dropping some items. The psychometric properties of CPD-REACTION reported in included studies showed that Cronbach *α* scores were very good, ranging from 0.62-0.91. However, few studies were designed to assess changes in intention (ie, scoring both pre- and postactivity), thus limiting the evidence regarding the responsiveness of the tool. Regarding behavioral intention to change, the mean difference of intention score was 0.54 SD 0.13 in the pre-post studies and the distribution of scores across all studies using CPD-REACTION ranged from 1.8-7. Although none of the studies fully met all MMAT criteria, none were rated “no” for any criteria. In all the design groups, all the criteria not rated “yes” were rated “not sure.”

### Significance and Comparison With Prior Work

First, the rapid adoption of CPD-REACTION across time, countries, and languages suggests that this instrument addresses the needs of CPD developers and that they seek not only validated assessment tools but also those that are informed by behavior change theories. Recent literature on this topic tends to suggest an increasing penetration of behavior change theories in the CPD developer community [2,76,77]. The use of behavior change theory has been frequently linked to effectiveness in systematic reviews of behavioral change interventions [76,77]. More recently, strategies have also focused not only on adopting new behaviors but also on abandoning low-value or harmful behaviors. However, few behavioral theories distinguish between behavior adoption and abandonment, including the theories on which CPD-REACTION is based [78]. Future research should distinguish between the two and develop theories that support both types of behavior change [79,80].

Second, physicians and nurses were the most represented health professionals. Most groups of participants engaging in CPD activities were interprofessional clinical teams, and 1 in 10 studies included managers or decision-makers among participants. This suggests that CPD designers are increasingly creating multidisciplinary training experiences to be shared with other stakeholders and professionals to enhance the relevance and impact of CPD [2,81]. Previous research has highlighted that including peer groups seems to be an effective approach to enhancing CPD activities and moving forward with professional practice change [82]. Future research should determine the effects of interprofessional participant groups or peer groups on CPD effectiveness.

Third, studies using an adapted version of CPD-REACTION reported Cronbach *α* ranging from .62 to .91, indicating that modified instruments perform well in terms of their psychometric properties. Other studies have reported psychometric values mentioned in the original version of CPD-REACTION. We observed that overall, the behavioral change intention scores reported ranged from 2.9 to 7. In pre-post studies, the mean difference in intention scores was 0.54 SD 0.13, and the distribution of scores across all constructs ranged from 1.8 to 7. Lower scores were observed when CPD-REACTION was used either only preactivity or only postactivity. Dissemination of the user manual will aid in the use of the tool to its best advantage. A lower score could also be because of the CPD topic being more controversial and thus less likely to be implementable. Overall, the adapted versions of CPD-REACTION reported Cronbach *α* values, indicating that the questionnaire had good internal consistency reliability. Furthermore, our results suggest that CPD-REACTION is adaptable to digital platforms, as two-thirds of the activities were web-based.

Fourth, using CPD-REACTION to measure construct scores, both pre- and post-CPD activity, is a helpful demonstration of the effect of CPD activities on behavioral intention and explanatory constructs. However, measuring learning outcomes for levels 3 and 4 of the Kirkpatrick model remains challenging. CPD-REACTION uses intention as a measure of behavioral intention; however, other measurement strategies are needed to directly measure behavior change. Although other outcomes such as “satisfaction of participants” were reported, the studies did not correlate these with the CPD-REACTION measures. In some studies, participants were contacted after 3 months or more to self-assess their behavior change [8]. The purpose of CPD-REACTION was not to measure its effects on patient outcomes, which is another important outcome of CPD. CPD-REACTION could be followed up by participant surveys to assess the longer-term impacts of participants’ behaviors on their practices or institutions and should use patient-reported measures. Some studies suggest that CPD programs should compare self-assessments, such as CPD-REACTION, with continuous formal participant multisource assessment by peers [73].

### Limitations

Our systematic review used diverse strategies to find studies that had used CPD-REACTION. However, we relied on the published results and did not contact the authors of the included studies. Thus, it is possible that we may have missed studies that were not published as well as items of interest in those we included. Owing to the large number of included studies, we had to organize the information into broad categories to increase the interpretability of the data.

### Conclusions

The CPD-REACTION questionnaire is a simple, relevant, and easy-to-use tool for assessing the effectiveness of CPD activities on health professionals’ behavioral intention and, as we have observed, to identify barriers and facilitators of behavior change. This tool has been used to evaluate CPD activities in a wide range of clinical topics and behaviors. However, most users do not measure intention both before and after the activity. Dissemination of a user manual will aid in the use of the tool to its best advantage. Further research should investigate the most effective way to adapt the CPD-REACTION questionnaire to various uses and contexts.

## Appendix

Multimedia Appendix 1 Search strategy.

Multimedia Appendix 2 Mixed Methods Appraisal Tool criteria quality assessment in the included studies.

Multimedia Appendix 3 Evolution of the number of published studies using CPD-REACTION questionnaire, 2014-2021.

## Abbreviations

CPD: continuing professional development
MeSH: Medical Subject Headings
MMAT: Mixed Methods Appraisal Tool
PICOS: Population, Intervention, Comparator, Outcomes, Study design
PRISMA: Preferred Reporting Items for Systematic Reviews and Meta-Analyses
PROSPERO: International Prospective Register of Systematic Reviews

## Data Availability

The data sets used and analyzed during this study are available from the corresponding author upon reasonable request.
