# Supplementary material for: Use of the CPD-REACTION Questionnaire to Evaluate Continuing Professional Development Activities for Health Professionals: Systematic Review
Source: JMIR Med Educ. 2022 May 2;8(2):e36948. doi: 10.2196/36948 (PMC9112082; doi:10.2196/36948)
Supplement: Multimedia Appendix 1 [file mededu_v8i2e36948_app1.docx]

**Appendix n° 1 : Search strategy**

- **Medline (2021-04-19)**

| **PICOS** | **Concept** | **Research strategy keywords** | **Research** | **# Results** |
| --- | --- | --- | --- | --- |
| Intervention | Education, Continuing (Controlled Vocabulary) | exp Education, Continuing/ | #1 | 61 675 |
|  | Questionnaires  (Controlled vocabulary) | "surveys and questionnaires"/ or self report/ or exp Program Evaluation/ | #2 | 583 466 |
|  | CPD-Reaction Questionnaire (Controlled vocabulary) | 1 and 2 | #3 | 7 759 |
|  | CPD-Reaction Questionnaire (Free text) | (("Continuing Professional Development" or "Continuous Professional Development" or "Continuing Professional Education" or "Continuous Professional Education" or "continuing education" or "Developpement professionnel continu" or "graduate training" or "Planned Behavior" or "behavioural intention*" or "Social Cognitive Theory") adj6 (questionnaire* or survey* or instrument* or assess* or evaluat* or tool*)).ti.  or (("Continuing Professional Development" or "Continuous Professional Development" or "Continuing Professional Education" or "Continuous Professional Education" or "continuing education" or "Developpement professionnel continu" or "graduate training" or "Planned Behavior" or "behavioural intention*" or "Social Cognitive Theory") adj6 (questionnaire* or survey* or instrument* or assess* or evaluat* or tool*)).ab.  or ( ("Continuing Professional Development" or "Continuous Professional Development" or "Continuing Professional Education" or "Continuous Professional Education" or "continuing education" or "Developpement professionnel continu" or "graduate training" or "Planned Behavior" or "behavioural intention*" or "Social Cognitive Theory") adj6 (questionnaire* or survey* or instrument* or assess* or evaluat* or tool*) ).kf. | #4 | 1 735 |
|  | CPD-Reaction Questionnaire (Free text) | ("CPD-Reaction" or "DPC-Reaction" or "CPD questionnaire*" or "12-item tool" or "12-item theory-based instrument" or "SCT questionnaire").ti.  or ("CPD-Reaction" or "DPC-Reaction" or "CPD questionnaire*" or "12-item tool" or "12-item theory-based instrument" or "SCT questionnaire").ab.  or ("CPD-Reaction" or "DPC-Reaction" or "CPD questionnaire*" or "12-item tool" or "12-item theory-based instrument" or "SCT questionnaire" ).kf. | #5 | 26 |
|  | CPD-Reaction Questionnaire (Free text) | 4 or 5 | #6 | 1 756 |
|  | Total | 3 or 6 | #7 | 9 186 |
|  | Filter for Date* | Limit 7 to yr="2014 -Current" | #8 | 1 968 |
| *We did this limitation because the CPD-Reaction Questionnaire was published in 2014. | | | | |

|  |
| --- |

- **Embase (2021-04-19)**

| **PICOS** | **Concept** | **Research strategy keywords** | **Research** | **# Results** |
| --- | --- | --- | --- | --- |
| Intervention | Education, Continuing (Controlled Vocabulary) | 'continuing education'/exp or 'continuing education provider'/exp OR 'Theory of Planned Behavior'/exp OR 'Social Cognitive Theory'/exp | #1 | 35 855 |
|  | Questionnaires  (Controlled vocabulary) | 'open-ended questionnaire'/exp OR 'structured questionnaire'/exp OR 'questionnaire'/de OR 'survey'/exp OR 'surveys'/exp | #2 | 778 820 |
|  | CPD-Reaction Questionnaire (Controlled vocabulary) | #1 and #2 | #3 | 3 653 |
|  | CPD-Reaction Questionnaire (Free text) | (("Continuing Professional Development" or "Continuous Professional Development" or "Continuing Professional Education" or "Continuous Professional Education" or "continuing education" or "Developpement professionnel continu" or "graduate training" or "Planned Behavior" or "behavioural intention*" or "Social Cognitive Theory") NEAR/6 (questionnaire* or survey* or instrument* or assess* or evaluat* or tool*)):ti,ab,kw | #4 | 3 028 |
|  | CPD-Reaction Questionnaire (Free text) | ("CPD-Reaction" or "DPC-Reaction" or "CPD questionnaire*" or "12-item tool" or "12-item theory-based instrument" or "SCT questionnaire"):ti,ab,kw | #5 | 40 |
|  | CPD-Reaction Questionnaire (Free text) | #4 or #5 | #6 | 3 061 |
|  | Total | #3 or #6 | #7 | 6 415 |
|  | Filter for Date* | (#3 OR #6) AND [18-3-2014]/sd | #8 | 2 247 |
| *We did this limitation because the CPD-Reaction Questionnaire was published in 2014. | | | | |

- **Web of Science (2021-04-19)**

| **PICOSS** | **Concept** | **Research strategy keywords** | **Research** | **# Results** |
| --- | --- | --- | --- | --- |
| Intervention | CPD-Reaction Questionnaire (Free text) | TS=(("Continuing Professional Development" or "Continuous Professional Development" or "Continuing Professional Education" or "Continuous Professional Education" or "continuing education" or "Developpement professionnel continu" or "graduate training" or "Planned Behavior" or "behavioural intention*" or "Social Cognitive Theory") NEAR/5 (questionnaire* or survey* or instrument* or assess* or evaluat* or tool*)) | #1 | 2 072 |
|  | CPD-Reaction Questionnaire (Free text) | TS=("CPD-Reaction" or "DPC-Reaction" or "CPD questionnaire*" or "12-item tool" or "12-item theory-based instrument" or "SCT questionnaire") | #2 | 29 |
|  | Total | #1 or #2 | #3 | 2 095 |
|  | Filter for Date* | #3 AND Timespan=2014-2021 | #4 | 985 |
| *We did this limitation because the CPD-Reaction Questionnaire was published in 2014. | | | | |

- **Eric-EBSCO (2021-04-19)**

| **PICOSS** | **Concept** | **Research strategy keywords** | **Research** | **# Results** |
| --- | --- | --- | --- | --- |
| Intervention | Education, Continuing (Controlled Vocabulary) | DE "Continuing Education" OR DE "Mandatory Continuing Education" OR DE "Professional Continuing Education" | #1 | 10 330 |
|  | Questionnaires  (Controlled vocabulary) | DE "Surveys" OR DE "Online Surveys" OR DE "Parent Surveys" OR DE "Student Surveys" OR DE "Questionnaires" OR DE "Program Evaluation" | #2 | 163 411 |
|  | CPD-Reaction Questionnaire (Controlled vocabulary) | S1 and S2 | #3 | 1 422 |
|  | CPD-Reaction Questionnaire (Free text) | TI ( ("Continuing Professional Development" or "Continuous Professional Development" or "Continuing Professional Education" or "Continuous Professional Education" or "continuing education" or "Developpement professionnel continu" or "graduate training" or "Planned Behavior" or "behavioural intention*" or "Social Cognitive Theory") N6 (questionnaire* or survey* or instrument* or assess* or evaluat* or tool*) )  OR AB ( ("Continuing Professional Development" or "Continuous Professional Development" or "Continuing Professional Education" or "Continuous Professional Education" or "continuing education" or "Developpement professionnel continu" or "graduate training" or "Planned Behavior" or "behavioural intention*" or "Social Cognitive Theory") N6 (questionnaire* or survey* or instrument* or assess* or evaluat* or tool*) )  OR SU ( ("Continuing Professional Development" or "Continuous Professional Development" or "Continuing Professional Education" or "Continuous Professional Education" or "continuing education" or "Developpement professionnel continu" or "graduate training" or "Planned Behavior" or "behavioural intention*" or "Social Cognitive Theory") N6 (questionnaire* or survey* or instrument* or assess* or evaluat* or tool*) ) | #4 | 1 171 |
|  | CPD-Reaction Questionnaire (Free text) | TI ( "CPD-Reaction" or "DPC-Reaction" or "CPD questionnaire*" or "12-item tool" or "12-item theory-based instrument" or "SCT questionnaire" )  OR AB ( "CPD-Reaction" or "DPC-Reaction" or "CPD questionnaire*" or "12-item tool" or "12-item theory-based instrument" or "SCT questionnaire" )  OR SU ( "CPD-Reaction" or "DPC-Reaction" or "CPD questionnaire*" or "12-item tool" or "12-item theory-based instrument" or "SCT questionnaire" ) | #5 | 3 |
|  | CPD-Reaction Questionnaire (Free text) | S4 or S5 | #6 | 1 174 |
|  | Total | S3 or S6 | #7 | 2 364 |
|  | Filter for Date* | Restriction Operators - Publication Date : 20140101-20211231 | #8 | 273 696 |
|  | Total with filter for date | S7 AND S8 | #9 | 288 |
| *We did this limitation because the CPD-Reaction Questionnaire was published in 2014. | | | | |

- **PsycInfo-Ovid (2021-04-19)**

| **PICOSS** | **Concept** | **Research strategy keywords** | **Research** | **# Results** |
| --- | --- | --- | --- | --- |
| Intervention | Education, Continuing (Controlled Vocabulary) | exp continuing education/ or exp planned behavior/ | #1 | 9 691 |
|  | Questionnaires  (Controlled vocabulary) | exp surveys/ or questionnaires/ or exp self-evaluation/ or exp program evaluation/ | #2 | 62 442 |
|  | CPD-Reaction Questionnaire (Controlled vocabulary) | 1 and 2 | #3 | 326 |
|  | CPD-Reaction Questionnaire (Free text) | (("Continuing Professional Development" or "Continuous Professional Development" or "Continuing Professional Education" or "Continuous Professional Education" or "continuing education" or "Developpement professionnel continu" or "graduate training" or "Planned Behavior" or "behavioural intention*" or "Social Cognitive Theory") adj6 (questionnaire* or survey* or instrument* or assess* or evaluat* or tool*)).ti.  or (("Continuing Professional Development" or "Continuous Professional Development" or "Continuing Professional Education" or "Continuous Professional Education" or "continuing education" or "Developpement professionnel continu" or "graduate training" or "Planned Behavior" or "behavioural intention*" or "Social Cognitive Theory") adj6 (questionnaire* or survey* or instrument* or assess* or evaluat* or tool*)).ab.  or ( ("Continuing Professional Development" or "Continuous Professional Development" or "Continuing Professional Education" or "Continuous Professional Education" or "continuing education" or "Developpement professionnel continu" or "graduate training" or "Planned Behavior" or "behavioural intention*" or "Social Cognitive Theory") adj6 (questionnaire* or survey* or instrument* or assess* or evaluat* or tool*) ).hw. | #4 | 1 115 |
|  | CPD-Reaction Questionnaire (Free text) | ("CPD-Reaction" or "DPC-Reaction" or "CPD questionnaire*" or "12-item tool" or "12-item theory-based instrument" or "SCT questionnaire").ti.  or ("CPD-Reaction" or "DPC-Reaction" or "CPD questionnaire*" or "12-item tool" or "12-item theory-based instrument" or "SCT questionnaire").ab.  or ("CPD-Reaction" or "DPC-Reaction" or "CPD questionnaire*" or "12-item tool" or "12-item theory-based instrument" or "SCT questionnaire" ).hw. | #5 | 7 |
|  | CPD-Reaction Questionnaire (Free text) | 4 or 5 | #6 | 1 120 |
|  | Total | 3 or 6 | #7 | 1 394 |
|  | Filter for Date* | Limit 7 to yr="2014 -Current" | #8 | 434 |
| *We did this limitation because the CPD-Reaction Questionnaire was published in 2014. | | | | |

- **Social Sciences Full Text-EBSCO (2021-04-19)**

| **PICOSS** | **Concept** | **Research strategy keywords** | **Research** | **# Results** |
| --- | --- | --- | --- | --- |
| Intervention | Education, Continuing (Controlled Vocabulary) | DE "Continuing education" | #1 | 412 |
|  | Questionnaires  (Controlled vocabulary) | (DE "Questionnaire design") OR (DE "Self-evaluation") | #2 | 8 044 |
|  | CPD-Reaction Questionnaire (Controlled vocabulary) | S1 and S2 | #3 | 13 |
|  | CPD-Reaction Questionnaire (Free text) | TI ( ("Continuing Professional Development" or "Continuous Professional Development" or "Continuing Professional Education" or "Continuous Professional Education" or "continuing education" or "Developpement professionnel continu" or "graduate training" or "Planned Behavior" or "behavioural intention*" or "Social Cognitive Theory") N6 (questionnaire* or survey* or instrument* or assess* or evaluat* or tool*) )  OR AB ( ("Continuing Professional Development" or "Continuous Professional Development" or "Continuing Professional Education" or "Continuous Professional Education" or "continuing education" or "Developpement professionnel continu" or "graduate training" or "Planned Behavior" or "behavioural intention*" or "Social Cognitive Theory") N6 (questionnaire* or survey* or instrument* or assess* or evaluat* or tool*) )  OR SU ( ("Continuing Professional Development" or "Continuous Professional Development" or "Continuing Professional Education" or "Continuous Professional Education" or "continuing education" or "Developpement professionnel continu" or "graduate training" or "Planned Behavior" or "behavioural intention*" or "Social Cognitive Theory") N6 (questionnaire* or survey* or instrument* or assess* or evaluat* or tool*) ) | #4 | 130 |
|  | CPD-Reaction Questionnaire (Free text) | TI ( "CPD-Reaction" or "DPC-Reaction" or "CPD questionnaire*" or "12-item tool" or "12-item theory-based instrument" or "SCT questionnaire" )  OR AB ( "CPD-Reaction" or "DPC-Reaction" or "CPD questionnaire*" or "12-item tool" or "12-item theory-based instrument" or "SCT questionnaire" )  OR SU ( "CPD-Reaction" or "DPC-Reaction" or "CPD questionnaire*" or "12-item tool" or "12-item theory-based instrument" or "SCT questionnaire" ) | #5 | 2 |
|  | CPD-Reaction Questionnaire (Free text) | S4 or S5 | #6 | 132 |
|  | Total | S3 or S6 | #7 | 145 |
|  | Filter for Date* | Restriction Operators - Publication Date : 20140101-20211231 | #8 | 658 365 |
|  | Total with filter for date | S7 AND S8 | #9 | 53 |
| *We did this limitation because the CPD-Reaction Questionnaire was published in 2014. | | | | |

- **CINAHL Plus with Full Text (2021-04-19)**

| **PICOSS** | **Concept** | **Research strategy keywords** | **Research** | **# Results** |
| --- | --- | --- | --- | --- |
| Intervention | Education, Continuing (Controlled Vocabulary) | (MH "Education, Continuing+") | #1 | 35 583 |
|  | Questionnaires  (Controlled vocabulary) | (MH "Questionnaires+") OR (MH "Program Evaluation") OR (MH "Surveys") OR (MH "Self Report+") | #2 | 595 235 |
|  | CPD-Reaction Questionnaire (Controlled vocabulary) | S1 and S2 | #3 | 4 764 |
|  | CPD-Reaction Questionnaire (Free text) | TI ( ("Continuing Professional Development" or "Continuous Professional Development" or "Continuing Professional Education" or "Continuous Professional Education" or "continuing education" or "Developpement professionnel continu" or "graduate training" or "Planned Behavior" or "behavioural intention*" or "Social Cognitive Theory") N6 (questionnaire* or survey* or instrument* or assess* or evaluat* or tool*) )  OR AB ( ("Continuing Professional Development" or "Continuous Professional Development" or "Continuing Professional Education" or "Continuous Professional Education" or "continuing education" or "Developpement professionnel continu" or "graduate training" or "Planned Behavior" or "behavioural intention*" or "Social Cognitive Theory") N6 (questionnaire* or survey* or instrument* or assess* or evaluat* or tool*) )  OR SU ( ("Continuing Professional Development" or "Continuous Professional Development" or "Continuing Professional Education" or "Continuous Professional Education" or "continuing education" or "Developpement professionnel continu" or "graduate training" or "Planned Behavior" or "behavioural intention*" or "Social Cognitive Theory") N6 (questionnaire* or survey* or instrument* or assess* or evaluat* or tool*) ) | #4 | 1 802 |
|  | CPD-Reaction Questionnaire (Free text) | TI ( "CPD-Reaction" or "DPC-Reaction" or "CPD questionnaire*" or "12-item tool" or "12-item theory-based instrument" or "SCT questionnaire" )  OR AB ( "CPD-Reaction" or "DPC-Reaction" or "CPD questionnaire*" or "12-item tool" or "12-item theory-based instrument" or "SCT questionnaire" )  OR SU ( "CPD-Reaction" or "DPC-Reaction" or "CPD questionnaire*" or "12-item tool" or "12-item theory-based instrument" or "SCT questionnaire" ) | #5 | 37 |
|  | CPD-Reaction Questionnaire (Free text) | S4 or S5 | #6 | 1 836 |
|  | Total | S3 or S6 | #7 | 6 304 |
|  | Filter for Date* | Restriction Operators - Publication Date : 20140101-20191231 | #8 | 2 089 |
| *We did this limitation because the CPD-Reaction Questionnaire was published in 2014. | | | | |

- **Academic Search Premier-EBSCO (2021-04-20)**

| **PICOSS** | **Concept** | **Research strategy keywords** | **Research** | **# Results** |
| --- | --- | --- | --- | --- |
| Intervention | Education, Continuing (Controlled Vocabulary) | DE "CONTINUING education" OR DE "COMPULSORY continuing education" OR DE "CONTINUING education of nurses" OR DE "CONTINUING medical education" OR DE "PLANNED behavior theory" | #1 | 24 252 |
|  | Questionnaires  (Controlled vocabulary) | DE "MEDICAL care surveys" OR DE "PATIENT surveys" OR DE "HOSPITAL patient surveys" OR DE "SURVEYS" OR DE "INTERNET surveys" OR DE "EMAIL surveys" OR DE "MAIL surveys" OR DE "QUESTIONNAIRE design" | #2 | 93 397 |
|  | CPD-Reaction Questionnaire (Controlled vocabulary) | S1 and S2 | #3 | 621 |
|  | CPD-Reaction Questionnaire (Free text) | TI ( ("Continuing Professional Development" or "Continuous Professional Development" or "Continuing Professional Education" or "Continuous Professional Education" or "continuing education" or "Developpement professionnel continu" or "graduate training" or "Planned Behavior" or "behavioural intention*" or "Social Cognitive Theory") N6 (questionnaire* or survey* or instrument* or assess* or evaluat* or tool*) )  OR AB ( ("Continuing Professional Development" or "Continuous Professional Development" or "Continuing Professional Education" or "Continuous Professional Education" or "continuing education" or "Developpement professionnel continu" or "graduate training" or "Planned Behavior" or "behavioural intention*" or "Social Cognitive Theory") N6 (questionnaire* or survey* or instrument* or assess* or evaluat* or tool*) )  OR SU ( ("Continuing Professional Development" or "Continuous Professional Development" or "Continuing Professional Education" or "Continuous Professional Education" or "continuing education" or "Developpement professionnel continu" or "graduate training" or "Planned Behavior" or "behavioural intention*" or "Social Cognitive Theory") N6 (questionnaire* or survey* or instrument* or assess* or evaluat* or tool*) ) | #4 | 1 850 |
|  | CPD-Reaction Questionnaire (Free text) | TI ( "CPD-Reaction" or "DPC-Reaction" or "CPD questionnaire*" or "12-item tool" or "12-item theory-based instrument" or "SCT questionnaire" )  OR AB ( "CPD-Reaction" or "DPC-Reaction" or "CPD questionnaire*" or "12-item tool" or "12-item theory-based instrument" or "SCT questionnaire" )  OR SU ( "CPD-Reaction" or "DPC-Reaction" or "CPD questionnaire*" or "12-item tool" or "12-item theory-based instrument" or "SCT questionnaire" ) | #5 | 32 |
|  | CPD-Reaction Questionnaire (Free text) | S4 or S5 | #6 | 1 871 |
|  | Total | S3 or S6 | #7 | 2 438 |
|  | Filter for Date* | Restriction Operators - Publication Date : 20140101-20191231 | #8 | 13 563 097 |
|  | Total with filter for date | S7 AND S8 | #9 | 1 062 |
| *We did this limitation because the CPD-Reaction Questionnaire was published in 2014. | | | | |

- **Google Scholar (2021-04-20)**

| **PICOSS** | **Concepts** | **Research strategy keywords** | **Research** | **# Results** |
| --- | --- | --- | --- | --- |
| Intervention | DPC-Reaction questionnaire | We have selected articles that quote the following article : Légaré F, Borduas F, Freitas A, Jacques A, Godin G, Luconi F, Grimshaw J and the CPD-KT team (2014) Development of a simple 12-item theory-based instrument to assess the impact of continuing professional development on clinical behavioral intentions. PLoS ONE 9(3): e91013. doi:10.1371/journal.pone.0091013 | #1 | 77 |
|  | DPC-Reaction questionnaire | We have selected articles that quote the following article : Légaré F, Borduas F, Jacques A, et al. Developing a theory-based instrument to assess the impact of continuing professional development activities on clinical practice : A study protocol. Implement Sci. 2011;6:17 | #2 | 34 |
|  | DPC-Reaction questionnaire | We have selected articles that quote the following article : Légaré F, Freitas A, Thompson-Leduc P, Borduas F, Luconi F, et al. (2015) The majority of accredited continuing professional development activities do not target clinical behavior change. | #3 | 63 |

- **Research gate (2021-04-20)**

| **PICOSS** | **Concepts** | **Research strategy keywords** | **Research** | **# Results** |
| --- | --- | --- | --- | --- |
| Intervention | DPC-Reaction questionnaire | We have selected articles that quote the following article : Légaré F, Borduas F, Freitas A, Jacques A, Godin G, Luconi F, Grimshaw J and the CPD-KT team (2014) Development of a simple 12-item theory-based instrument to assess the impact of continuing professional development on clinical behavioural intentions. PLoS ONE 9(3): e91013. doi:10.1371/journal.pone.0091013 | #1 | 56 |
|  | DPC-Reaction questionnaire | We have selected articles that quote the following article : Légaré F, Borduas F, Jacques A, et al. Developing a theory-based instrument to assess the impact of continuing professional development activities on clinical practice : A study protocol. Implement Sci. 2011;6:17 | #2 | 24 |
|  | DPC-Reaction questionnaire | We have selected articles that quote the following article : Légaré F, Freitas A, Thompson-Leduc P, Borduas F, Luconi F, et al. (2015) The majority of accredited continuing professional development activities do not target clinical behaviour change. | #3 | 36 |

- **Web of Science (2021-04-20)**

| **PICO** | **Concepts** | **Research strategy keywords** | **Research** | **# Results** |
| --- | --- | --- | --- | --- |
| Intervention | DPC-Reaction questionnaire | We have selected articles that quote the following article: Légaré F, Borduas F, Freitas A, Jacques A, Godin G, Luconi F, Grimshaw J and the CPD-KT team (2014) Development of a simple 12-item theory-based instrument to assess the impact of continuing professional development on clinical behavioural intentions. PLoS ONE 9(3): e91013. doi:10.1371/journal.pone.0091013 | #1 | 44 |
|  | DPC-Reaction questionnaire | We have selected articles that quote the following article : Légaré F, Borduas F, Jacques A, et al. Developing a theory-based instrument to assess the impact of continuing professional development activities on clinical practice : A study protocol. Implement Sci. 2011;6:17 | #2 | 15 |
|  | DPC-Reaction questionnaire | We have selected articles that quote the following article : Légaré F, Freitas A, Thompson-Leduc P, Borduas F, Luconi F, et al. (2015) The majority of accredited continuing professional development activities do not target clinical behaviour change. | #3 | 29 |
