# Supplementary figures and images for: Use of the CPD-REACTION Questionnaire to Evaluate Continuing Professional Development Activities for Health Professionals: Systematic Review
Source: JMIR Med Educ. 2022 May 2;8(2):e36948. doi: 10.2196/36948 (PMC9112082; doi:10.2196/36948)

## Appendix n° 2: MMAT criteria quality assessment in the included studies n=52

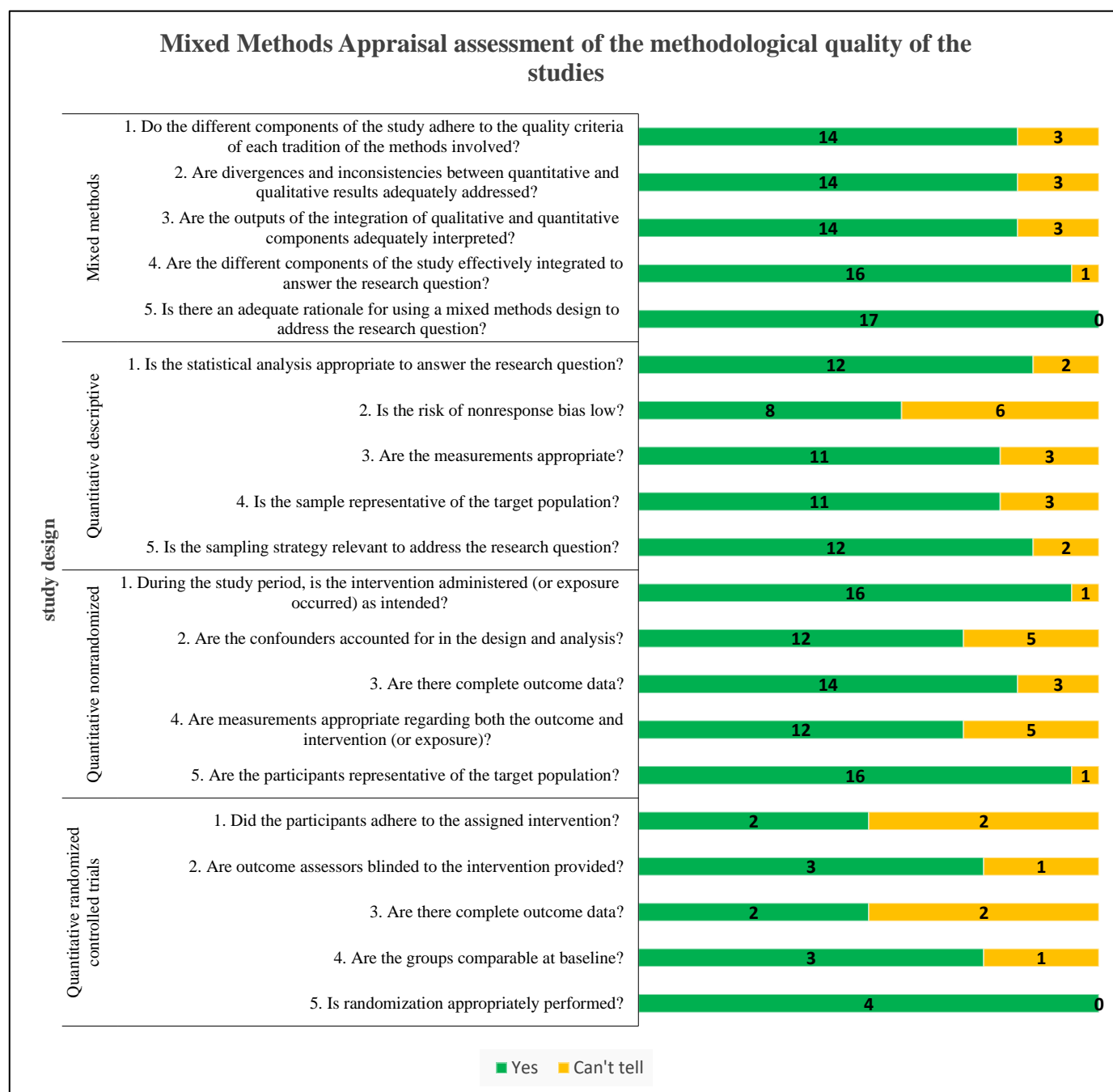

Supplement: Multimedia Appendix 2 [file mededu_v8i2e36948_app2.pdf]

## Slide 1
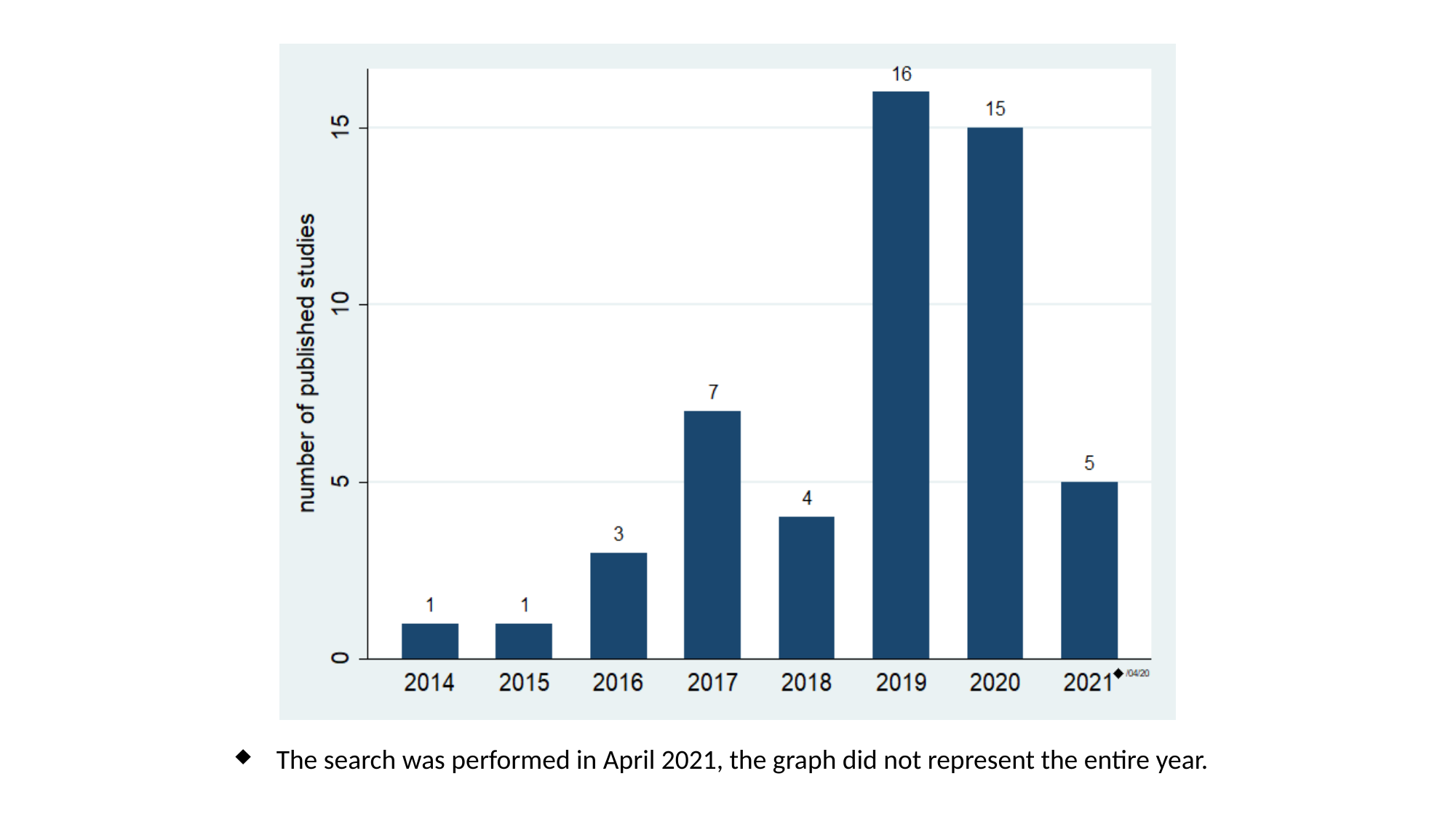

The search was performed in April 2021, the graph did not represent the entire year.

Supplement: Multimedia Appendix 3 [file mededu_v8i2e36948_app3.pptx]
